# 

**Published:** 1886-12

**Authors:** 


					﻿D E C E M B E R . 1 8 8 6
OLD SERIES
Vol. XXV
_ & 1855 O'
fMJW SERIE^
Vol. HI.—No. 10-/

■edigal^Surw
» JOURNA^B

11 w.f.westmore£andjO
HV.M.MILLER. M.D.LL .DjE
JAMES A.GRAY. MJJ.’l
Published By james rharrison&co
IB? _______________________«flTLANTA,GA. JbMj
SUBSCRIPTION: $2.60 A YEAR.
				

